# A Modified Approach in Lip Repositioning Surgery for Excessive Gingival Display to Minimize Post-Surgical Relapse: A Randomized Controlled Clinical Trial

**DOI:** 10.3390/diagnostics13040716

**Published:** 2023-02-14

**Authors:** Reham N. AlJasser

**Affiliations:** Department of Periodontics and Community Dentistry, College of Dentistry, King Saud University, Riyadh 11545, Saudi Arabia; raljasser@ksu.edu.sa

**Keywords:** GD, lip repositioning, lip surgery, gummy smile, lip length, LipStaT^®^

## Abstract

Lip repositioning surgeries are performed to treat patients with excessive GD (EGD). This study aimed to explore and compare the long-term clinical results and stability following the modified lip repositioning surgical technique (MLRS) with the addition of periosteal sutures compared to the conventional lip repositioning surgery (LipStaT^®^) in order to address EGD. A controlled clinical trial with female participants (*n* = 200) intended to improve their gummy smile were divided into control (*n* = 100) and test (*n* = 100) groups. The gingival display (GD), maxillary lip length at rest (MLLR), and maxillary lip length at maximum smile (MLLS) were measured at four time intervals (Baseline; 1 Month; 6 Months, and 1 Year) in millimeters (mm). Data were analyzed by *t*-tests, Bonferroni-test, and regression analysis using SPSS software. At the one-year follow-up, GD for the control and test groups were 3.77 + 1.76 mm and 2.48 + 0.86 mm, respectively, and their comparisons showed that GD was considerably lower (*p* = 0.000) in the test group compared to the control group. The MLLS measurements taken at baseline, one-month, six-month, and one-year follow-up showed no significant differences (*p* > 0.05) between the control and test groups. At baseline, one-month, and six-month follow-up, the mean and standard deviation for the MLLR were almost similar, with no statistically significant difference (*p* = 0.675). The MLRS is a successful and viable treatment option for the treatment of patients with EGD. The current study showed stable results and no recurrence with MLRS until the one-year follow-up compared to LipStaT^®^. With the MLRS, a 2 to 3 mm decline in EGD is usually to be expected.

## 1. Introduction

People are increasingly being inspired to get more corrective and cosmetic surgeries as an esthetic smile becomes a more fundamental component of what it means to be beautiful. There are several variables that affect how appealing and esthetic a smile is [1]. Esthetic perception varies according to cultural, societal, environmental, and individual factors like experience and educational level [2]. Previous studies have shown that a smile with less gingival display (GD) is viewed as more beautiful, with dental professionals being more critical of gingival presentation than laypeople [3].

According to research by several authors, the ideal GD ranges from 1 to 3 mm [3,4]. While many factors influence how pleasant a smile is seen, excessive GD (EGD), sometimes referred to as a gummy smile, is regarded as a key factor in smile analysis and one of the main issues connected to an unsatisfactory dental smile [5]. A full, lively smile with an excess of more than 2–4 mm of gingival show is considered to have EGD [3,4,5]. When there is lip hypermobility, this may be more obvious. In a typical smile, at least 50% of patients present some type of GD (GD). However, up to 76% of all patients may display exaggerated or forced smile patterns [6]. In a “normal” smile, there should be 1–2 mm of GD between the gingival margin of the anterior central incisors and the inferior border of the upper lip. Conversely, laypeople and general dentists consider an excessive gingiva-to-lip distance of 4 mm or more to be “unattractive” [7,8].

EGD can have a number of etiologies, such as gingival enlargements, bony maxillary excess, inadequate maxillary lip length, hypermobile upper lip, and extra bone in the maxilla [4]. Therefore, the primary etiology or the combination of etiologies identified in each instance should be the focus of the therapeutic strategy. Normal upper lip translation during a dynamic smile typically ranges from 4–6 mm from rest. Clinical evaluation using lip translation from the relaxed posture to the widest smiling position can identify a hypermobile upper lip. A surgically predictable method known as lip repositioning surgery (LRS) is one of the therapy options available to rectify this excessive translation [9,10]. Therefore, it is absolutely crucial for dental practitioners to correctly identify the etiology before beginning any type of treatment to improve a patient’s smile.

In order to move the lip to a lower position throughout the healing process, a strip of mucosa from the maxillary vestibule must first be removed through a partial thickness incision. Then the lip mucosa must be stitched to the mucogingival line [6,9,11,12]. With this surgical procedure, numerous reports have shown positive results with good esthetic outcomes [12]. A significant percentage of relapse has, however, been seen in some cases six months to a year after the surgery. To prevent the lip muscle from relapsing into its original position, several articles [11,12] recommend removing the attachment. This may help reduce the flap strain during suturing. Utilizing an alloplastic or autogenous separator is another way to stop the muscles that control smiling from reattaching [13]. This spacer is positioned nasally between the lip’s elevator muscles and the anterior nasal spine, preventing the relocated lip from advancing. 

Furthermore, rhinoplasty and lip realignment have also been proposed [14]. There are case reports of frenectomy with crown lengthening, as well as lip repositioning combined with depigmentation and lengthening of the crown. The combination of lip realignment and crown lengthening has also been done using a laser [11,12,13,14,15,16].

Recently, several case studies have described some modifications to the traditional/conventional lip repositioning surgery (LipStaT^®^) for treating EGD; however, studies with a good number of patients and long-term follow-up for the modified lip repositioning surgical technique (MLRS) are lacking [6,17,18,19]. Only one study by Al Jasser et al., 2021 [20], on a twin population utilizing the MLRS, was found in the literature. The results of that study showed promising results with MLRS; however, due to the low sample size, the authors recommended future studies with a larger sample size. Thus, the present study aimed to explore and compare the long-term qualitative and quantitative results in clinical changes following an MLRS procedure that used periosteal sutures to secure the new lip position compared to the classic LRS in order to address EGD.

## 2. Materials and Methods

### 2.1. Ethical Approval

At the College of Dentistry Research Center, King Saud University, Riyadh, Saudi Arabia, the Institutional Review Board (IRB) and Institutional Committee of Research Ethics approved and registered (Approval/Registration no. E-18-113207) the study for ethical reasons. The 2013 revision of the 1975 Helsinki Declaration was followed when conducting the study.

### 2.2. Study Participants

The target audience included female subjects who intended to improve their gummy smile. After choosing an appropriate target population for the study, the communication was tailored to each participant to convey all pertinent details about the proposed surgical procedures’ measures and associated clinical parameters, as well as the study’s objectives, design, risks, and potential benefits. The lead researcher contacted the study’s target population and asked for their participation. People who expressed interest in the study were then evaluated for the inclusion criteria, which stipulated that the subjects had to be (i) adults aged 18 or older, (ii) seeking treatment at the periodontology clinic located at the College of Dentistry at King Saud University, Riyadh, Saudi Arabia. (iii) Systemically healthy or eligible for periodontal surgical operations (ASA I & II), (iv) reported a wish to enhance the esthetics of their gummy smile/EGD. The study excluded people diagnosed with bony maxillary excess or with a history of receiving face Botox or filler injections. Those who met all the criteria were chosen to take part in the study. Before the study began, the informed consent forms were signed by all eligible subjects.

### 2.3. Design, Randomization, and Calibration of the Study

A single-centered, single-blinded, randomized, controlled clinical trial design was used in this investigation. Block randomization employed a straightforward randomization technique. It was simple to put into practice, and the allocation of treatment for the subjects was totally random. The subjects were kept in the dark about which treatment (LipStaT^®^/MLRS) each person would receive. This process was employed to avoid bias in research findings. The lead investigator (R.N.A.) performed all surgical procedures and examinations for calibration.

### 2.4. Selection and Calculation of Sample Sizes

The final sample size of 200 patients, 100 patients each in the control and test group, was calculated by the G Power^®^ software with confidence level of 95% and moderate effect size.

### 2.5. Examination and Diagnosis

Review of the participants’ family and medical histories and examinations within and outside the mouth was completed prior to commencement of the procedure. An orthodontist conducted a facial and skeletal investigation to rule out the presence of bony maxillary excess or skeletal deformity. Additionally, the clinical attachment level, bleeding index, plaque index, and keratinized tissue width was measured as part of the periodontal examination. According to Marcuschamer et al., the presence of altered passive eruption was ruled out by measuring the dimensions and ratio of the maxillary anterior teeth as well as the Zenith from the first molar to the first molar in the adjacent quadrant to make sure all teeth fell within normal dimensions [21]. Based on a fresh series of intra-oral peri-apical radiographs taken from the first molar to the last tooth on the opposing side, the entire bone level was evaluated. To rule out a short lip as the cause, the length of the maxillary lip length at rest (MLLR) was measured. A whole dynamic smile was used to quantify the GD on each tooth from the first molar to the first molar at three different sites for each anterior tooth. Using William’s periodontal probe, all measurements were taken and recorded to the nearest millimeter (Hu-Friedy Co., Chicago, IL, USA). The three-time readings’ mean served as the foundation for the final measurement.

### 2.6. Assessment and Measurement of GD, Maxillary Lip Length, and Mobility

**Figure 1:**
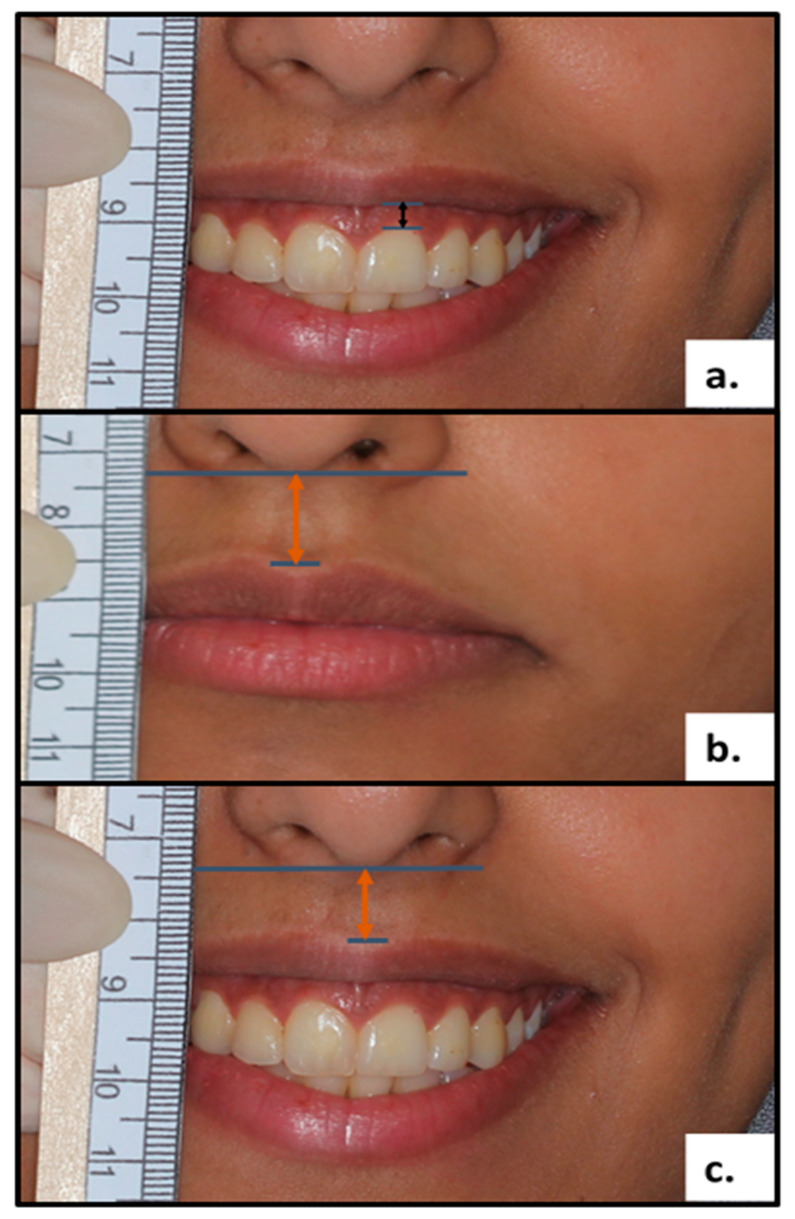
(**a**) Clinical Image of custom-made ruler used to measure gingival display (GS) as the vertical double arrow corresponds to GS measurement.; (**b**) clinical Image of custom-made ruler used to measure maxillary lip length at rest (MLLR) as the vertical double arrow corresponds to MLLR.; (**c**) clinical Image of custom-made ruler used to measure maxillary lip length at maximum smile (MLLS) as the vertical double black arrow corresponds to MLLS.

Each participant was seated upright while measurements were taken with a disposable 15 cm marked ruler. The GD over the maxillary right central incisor was measured using a specially manufactured millimeter ruler while the patient smiled the widest (Figure 1a). The distance between the sub-nasal and the most inferior part of the lip at the midline in the resting position and in maximum smile was used to determine the MLLR and maxillary lip length at maximum smile (MLLS) (Figure 1b,c). Measurements less than 20 mm were determined to be the short upper lip in the current study based on earlier data that stated the normal average lip length is 21.2 ± 2.4 mm to 23.4 ± 2.5 mm [22,23]. Finally, the amount of translation of the inferior border of the lip from the rest position at maximal smile was used to measure lip mobility. Hypermobile lip was diagnosed whenever translation exceeded 6 mm [12]. The same calibrated periodontist took all measurements at the baseline, one-month, six months, and one-year post-surgical follow-up.

### 2.7. Lip Repositioning Surgery Performed for the Control Group

The LipStaT^®^ method, as described by Bhola et al., was used in this group’s surgical protocol [12]. Before surgery, the participants were told to rinse for one minute with 0.12% chlorhexidine. Local infiltration (2% lidocaine with 1:50.000 epinephrine in the buccal vestibule) was used to produce anesthesia. A surgical marker was used to outline the boundaries of the surgical incision region. Based on the horizontal expansion of the dynamic grin, the inferior border was 1 mm coronal to the mucogingival junction and extended to the first molar area bilaterally. Based on a 2:1 ratio of vertical extension being twice the measurement of EGD at a full dynamic smile, the height of the superior incision was measured as 15 mm within the vestibule. Superior and inferior incisions were made with a scalpel blade number 15 and linked bilaterally by two vertical incisions. A partial thickness dissection was used to remove the strip of the indicated mucosa, exposing the fascia of the connective tissue beneath. When necessary, all salivary glands and frenal attachments were removed (Figure 1b). The surgical site was then properly closed using continuous interlocking sutures made of polypropylene 4/0 (PROLENE^®^ Polypropylene Suture, Ethicon US, LLC, Irvine, CA, USA) that were started on one side of the incision and ended on the opposite side. The new mucosal boundary to the gingiva was stabilized in its new place using this suture.

### 2.8. Surgical Modification Performed for Test Group

**Figure 2:**
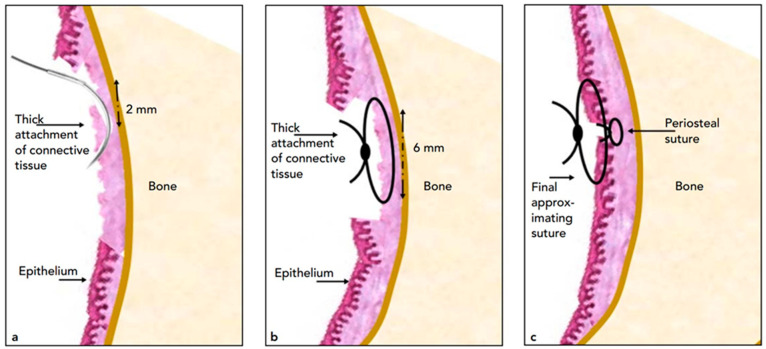
Schematic drawings portraying the periosteal suturing utilized. (**a**) The needle is inserted, starting 2 mm coronal to the base of the thick connective tissue attachment or frenal attachment, then the needle is slid apically, passing the attachment. (**b**) Sliding the needle up to 6 mm and tying a knot creates a simple interrupted suture. (**c**) The suture is intended to move and stabilize the thick connective tissue attachments in a more coronal position.

The test group underwent the same surgical approach, except that a periosteal simple interrupted suture was put in place prior to the continuous interlocking sutures. This vertical simple, interrupted suture was used in locations with strong connective tissue or frenal attachments. It was placed by commencing the needle 2 mm coronal to the base of the connection and moving it apically by crossing the connective tissue attachment up to 6 mm before tying the knot. The thick connective tissue attachments were supposed to be moved and stabilized by this suture in a more coronal position. Vicryl 4-0 resorbable sutures were used for all periosteal sutures (VICRYL RAPIDE™ (polyglactin 910) Suture, Ethicon US, LLC, Irvine, CA, USA). Per surgery site, 3 to 4 periosteal sutures were typically inserted (Figure 2). Finally, the same skilled periodontist conducted all of the procedures.

### 2.9. Post-Surgery Instructions

Analgesics (acetaminophen 750 mg/ibuprofen 400 mg alternating dosage every 4 h) were given to the participants for 2 days, and they were also told to rinse twice daily for 10 days with 0.12% chlorhexidine. They were told to use cold packs, stick to soft foods for the first week, prevent any other mechanical trauma to the surgical sites, and limit lip movement for the first two weeks after the procedure.

### 2.10. Follow-Up Visits

Each participant had the following post-operative appointments: weekly for the first four weeks, followed by appointments at one month, six months, and one year. Professional plaque control and a review of oral hygiene recommendations were done at each subsequent session. In the same way as the baseline, all clinical measurements were taken. When it was practical, digital photographs were taken to aid in monitoring further changes to the smile.

### 2.11. Data Analysis

The IBM SPSS version 21.0 was used to examine the data that had been gathered. For both the test and control groups, descriptive statistics were used to evaluate the outcome variables, which included the average GD, MLLR, and MLLS. Since the variables were measured in numerical form, calculating means and standard deviations was necessary for the descriptive analysis. The mean values of the quantitative outcome variables were compared using the Student’s *t*-test for independent samples. The mean values of the quantitative outcome variables at four different time intervals in the control and test groups were compared using repeated measures analysis of variance. To compare the mean values at the four time points, post-hoc multiple comparisons with the Bonferroni test were used. The significance level for all analyses was set at 0.05.

## 3. Results

After giving written agreement, 200 female patients—100 in the control group and 100 in the test group—took part in the study. The comparison of the means of the GD measured in millimeters (mm) for the participating patients’ control and test groups at four distinct time intervals is shown in Table 1. At the one-year follow-up, the GD’s mean and standard deviation for the control and test groups were 3.77 ± 1.76 mm and 2.48 ± 0.86 mm, respectively. At the one-year follow-up, the comparisons showed that the GD was considerably lower (*p* = 0.000) in the test group than in the control group.

Table 2 compares the participants in the control and test groups’ average MLLS throughout four different time periods. For the measurements taken at baseline, one-month, six-month, and one-year follow-up, there were statistically no differences between the control and test groups. However, the mean difference of (0.845 mm) was the highest between the mean for the control (10.58 ± 1.01 mm) and test (9.73 + 1.68 mm) groups at a one-year follow-up.

The comparison of the mean MLLR among the control and test groups at four different time periods is shown in Table 3. At baseline, one-month, and six-month follow-up, the mean and standard deviation for the MLLR were almost similar, with no statistically significant difference (*p* = 0.675). However, the MLLR for the control group (12.96 ± 1.98 mm) was lower than that for the test group (13.17 ± 2.14 mm), though statistically non-significant.

**Figure 3:**
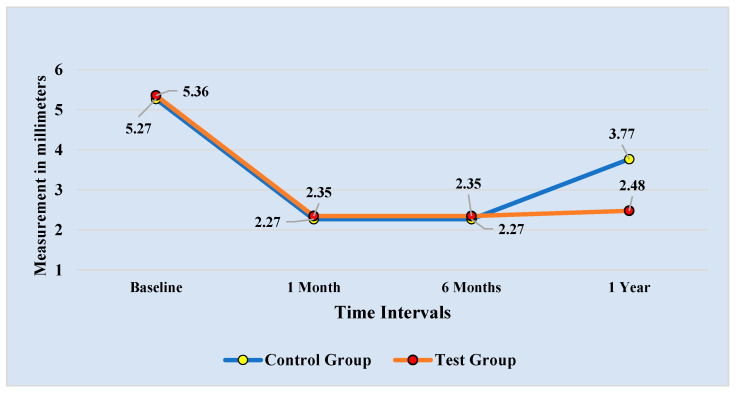
Comparison of the Gingival Display recorded at 4 time intervals.

Results for the repeated measurements and Bonferroni pairwise comparisons of the GD at the four time intervals for the control and test groups are shown in Figure 3 and Table 4, respectively. The measurements were taken one month, six months, and one year after the baseline readings showed a drop in the GD. The GD did, however, tend to rise significantly from the six-month reading at the one-year checkpoint for the control group, as opposed to the test group, where a very slight, non-significant rise was observed.

**Figure 4:**
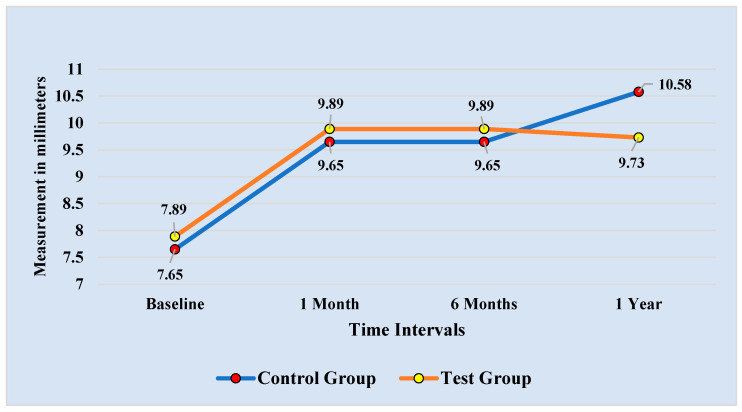
Comparison of Upper Lip Length at Maximum Smile recorded at 4 time intervals.

Results of repeated measures and Bonferroni pairwise comparisons of the MLLS at the four time intervals for the control and test groups are shown in Figure 4 and Table 5, respectively. The findings showed a non-significant increase in the MLLS from the baseline readings to the follow-up measurements after one month, six months, and one year. The MLLS for the control group, however, tended to rise from the 6-month reading at the one-year follow-up, in contrast to the test group, where a very slight decline was seen.

**Figure 5:**
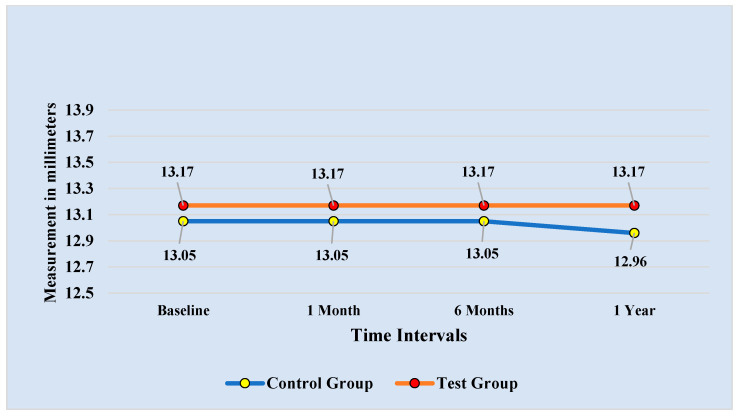
Comparison of Upper Lip Length at Rest recorded at 4 time intervals.

The results of the repeated measurements and Bonferroni pairwise comparisons of the MLLR at the four time intervals for the control and test groups are shown in Figure 5 and Table 6, respectively. The findings showed no statistically significant changes in the MLLR from the baseline readings to the follow-up measures at one month, six months, and one year. At the one-year follow-up, the MLLR did, however, tend to fall from the 6-month value for the control group only.

## 4. Discussion

Numerous studies have been conducted on LRS for excessive GD with minimal post-surgery relapse, and it has been noted that different populations have diverse responses to various surgical procedures [12,24,25]. This present study aimed to assess and contrast the relapse in relation to LipStaT^®^ surgery versus the MLRS surgical technique utilizing the periosteal sutures to secure the new lip position at four different time intervals (Baseline; one month, six months, and one-year, post-surgery). The findings of the present study showed that there was a significant variation in the amount of GD between the control and test groups, with the GD significantly lower than the control. The results also revealed minimal non-significant differences between the MLLS and MLLR. With the MLRS surgical technique employed for correcting excessive GD among the test group subjects, the GD was improved markedly, while there was no change in the upper lip length or profile at maximum smile and rest.

Since the 1970s, LRS has been referred to as a viable and conservative surgical option among the various treatment options (orthognathic surgery, botulin toxin injections, lip repositioning surgery, and/or the combination of therapies) for the correction of excessive GD caused by non-dentoalveolar etiologies [12,25,26]. The surgical method was generally carried out according to standard practice in the trials, with a first partial-thickness incision made around the mucogingival junction and a second one made 10 mm apically [12,20,26]. Although some studies performed full-thickness incisions and myotomy, the muscle fibers were preserved by only removing a thin layer of soft tissue in between the incisions [25]. Absorbable (Vicryl 4/0) suture material was most frequently utilized. Follow-ups lasted from one to twelve months, and 0.12% chlorhexidine rinse and anti-inflammatory drugs were often used as postoperative pharmaceutical therapy [12,25,26,27].

The process for LRS has undergone various modifications over the years, ranging from traditional surgical excision to diode laser procedures [12,25,26,27,28]. However, reducing smile muscle contractions was always the fundamental goal of all the treatments. To achieve this condition, partial-thickness elliptical incisions are used to remove a band of mucosa from the buccal vestibule depth, which is 3–4 mm above the gingival margin of the maxillary teeth. In terms of correcting EGD, the procedure produced respectable short-term results [12]. Although significant relapse percentages have been observed, which is thought to be the main drawback of this surgical approach, its efficacy is debatable [25]. The LRS-related relapse is often observed in the first 6 to 8 weeks for the vast majority of cases. However, some have reported it as late as 6 months or a year later. In 8–25% of the cases that were treated, relapse was discovered [18,25]. It is critical to look into the predictability of LRS in light of the growing number of technique variations and higher patient expectations that exist today.

The approach being used in a case with insufficient keratinized attached gingiva, cutting into the keratinized attached gingiva, deeply slicing into the connective tissue and muscle fibers, and/or situations with excessive muscle pull can all contribute to relapses [17]. The true nature of relapse in relation to the procedure is difficult to ascertain; however, the phenomena may be partially explained by muscle memory attempting to resume its preoperative activity [17,18]. Nevertheless, it is difficult to ascertain if this drop persists at levels close to baseline due to the absence of follow-ups longer than 12 months. Case reports make up the majority of literature records reporting long-term follow-ups of more than 12 months with LRS. After 2–4 years, acceptable stability with a minor relapse has been documented [18,25]. 

Additionally, it was noted in the reports that combining LRS with various adjunct medicines improved treatment outcomes and predictability [18,20,25]. The use of a scalpel in conventional LRS has been referenced in a number of research. In Rao et al. [6], the labial frenum was not touched during a LipStaT^®^ surgical lip repositioning procedure, which resulted in recurrence. Another study by Dayakar et al. [19] that included the labial frenum as part of the usual surgical LRS procedure revealed a complete relapse after 12 months. Relapses can be treated by either returning to the surgical site to incise additional mucosa as necessary or, as some articles have indicated, by administering Botox injections [18,25]. The surgical procedure used in this investigation was a straightforward modification to the classic LRS approach, which produces outstanding results. Compared to the control group, which underwent the LipStaT^®^ approach, there was no difference in the GD for the test group using the MLRS technique at the one-year follow-up.

Many changes to the procedure have been made and adopted since the invention of LRS. All of these adjustments were made with the goal of preventing the primary LRS-related recurrence problem [24,25,26,27,28,29]. Although few studies have looked at the long-term effects of LRS with myotomy, it was recommended as a method to prevent relapse and maintain stable results [18,25]. With only minor to moderate relapses, vestibuloplasty and muscle release are equally effective methods for treating EGD. This technique’s main benefit is that it may be quickly undone by vestibular deepening if the patient is unhappy with the results or repeated in the event of a recurrence [27]. It is also feasible to utilize a trial step after the measurements in which just sutures are used (no actual cutting) to give the patient an idea of what to expect from the ultimate outcome. Miskinyar changed the initial method into a myectomy and partial excision of the levator labii superioris rather than a complete separation from the bone due to the frequency of relapse [28]. This resection was thought to lower the likelihood of relapse.

In the long run, with LRS, it appears reasonable to say that the clinician should anticipate some degree of relapse following the surgery. Therefore, the patient should get education and information about the likelihood of relapse at the treatment planning stage. In addition, the perception and satisfaction of the patient should be taken into consideration in these situations. This fact suggests that for more predictable and stable results, the surgery should be combined with additional techniques such as restorative procedures, botulin toxin injections, or reconstructive periodontal surgeries. Botox injections have been proposed as a potential remedy for relapsed instances. Botox works by inhibiting muscle action, although the effects of using botulinum toxin are only temporary (6–7 months) [29]. By paralyzing the muscles during the healing process, Botox injections may be a helpful adjuvant in improving and stabilizing the effects of LRS and Botox as a pretreatment. Botox, in combination with LRS to lessen muscular strain and, hence, lessen the risk of recurrence, is a possible research issue that should be investigated but has not yet been studied, to the investigator’s knowledge. To strengthen the confidence in the current evidence, lengthier follow-ups and clinical investigations combining LRS with other methods and procedures should be carried out.

## 5. Conclusions

The modified lip repositioning surgical technique (MLRS) employed in the current study is a successful and viable therapy option for treating people with excessive GD compared to the classic/traditional LipStaT^®^ surgical method. The outcomes of the current study showed stable results and no recurrence with MLRS until the one-year follow-up, in contrast to the LipStaT^®^. With the MLRS, a 2 to 3 mm decline in EGD is usually to be expected.

## Figures and Tables

**Table 1:** Descriptive Statistics and Independent Samples *t*-Test for Gingival Display *, among the Control and Test Groups (*n* = 200).

Measurement Timeline	Patients (*n* = 200)	Mean	Std. Deviation	Std. Error Mean	Mean Difference	** Sig. (2-Tailed)
Base Line	Control (*n* = 100)	5.273	0.756	0.075	−0.095	0.049
	Test (*n* = 100)	5.369	0.872	0.087		
One Month	Control (*n* = 100)	2.274	0.756	0.075	−0.076	0.500
	Test (*n* = 100)	2.351	0.845	0.084		
Six Months	Control (*n* = 100)	2.274	0.756	0.075	−0.076	0.500
	Test (*n* = 100)	2.351	0.845	0.084		
One Year	Control (*n* = 100)	3.774	1.760	0.176	1.288	0.000
	Test (*n* = 100)	2.486	0.863	0.086		

* Gingival Display was recorded in millimeters (mm); ** *p*-value was considered significant at *p* ≤ 0.05.

**Table 2:** Descriptive Statistics and Independent Samples *t*-Test for the Upper Lip Length * at Maximum Smile, among the Control and Test Groups (*n* = 200).

Measurement Timeline	Patients (*n* = 200)	Mean	Std. Deviation	Std. Error Mean	Mean Difference	** Sig. (2-Tailed)
Base Line	Control (*n* = 100)	7.657	1.453	0.145	−0.238	0.277
	Test (*n* = 100)	7.895	1.632	0.163		
One Month	Control (*n* = 100)	9.657	1.453	0.145	−0.238	0.277
	Test (*n* = 100)	9.895	1.632	0.163		
Six Months	Control (*n* = 100)	9.657	1.453	0.145	−0.238	0.277
	Test (*n* = 100)	9.895	1.632	0.163		
One Year	Control (*n* = 100)	10.581	1.013	0.101	0.845	0.411
	Test (*n* = 100)	9.735	1.689	0.168		

* Upper Lip Length was recorded in millimeters (mm); ** *p*-value was considered significant at *p* ≤ 0.05.

**Table 3:** Descriptive Statistics and Independent Samples *t*-Test for the Upper Lip Length * at Rest, among the Control and Test Groups (*n* = 200).

Measurement Timeline	Patients (*n* = 200)	Mean	Std. Deviation	Std. Error Mean	Mean Difference	** Sig. (2-Tailed)
Base Line	Control (*n* = 100)	13.056	1.993	0.199	−0.122	0.675
	Test (*n* = 100)	13.179	2.146	0.214		
One Month	Control (*n* = 100)	13.056	1.993	0.199	−0.122	0.675
	Test (*n* = 100)	13.179	2.146	0.214		
Six Months	Control (*n* = 100)	13.056	1.993	0.199	−0.122	0.675
	Test (*n* = 100)	13.170	2.146	0.214		
One Year	Control (*n* = 100)	12.964	1.986	0.198	−0.214	0.464
	Test (*n* = 100)	13.179	2.146	0.214		

* Upper Lip Length was recorded in millimeters (mm); ** *p*-value was considered significant at *p* ≤ 0.05.

**Table 4:** Bonferroni pairwise comparisons of the Gingival Display (millimeters) at different time intervals for the participating subjects (*n* = 100).

		Control Group			Test Group		
Time Intervals	Compared to;	Mean Difference	Std. Error	* Sig.	Mean Difference	Std. Error	* Sig.
Baseline	1 Month	3.000 *	0.000	0.000	3.019 *	0.044	0.000
	6 Months	3.000 *	0.000	0.000	3.019 *	0.044	0.000
	1 Year	1.500 *	0.151	0.000	2.884 *	0.050	0.000
1 Month	Baseline	−3.000 *	0.000	0.000	−3.019 *	0.044	0.000
	6 Months	0.000	0.000		0.000	0.000	
	1 Year	−1.500 *	0.151	0.000	−0.135 *	0.022	0.000
6 Months	Baseline	−3.000 *	0.000	0.000	−3.019 *	0.044	0.000
	1 Month	0.000	0.000		0.000	0.000	
	1 Year	−1.500 *	0.151	0.000	−0.135 *	0.022	0.000
1 Year	Baseline	−1.500 *	0.151	0.000	−2.884 *	0.050	0.000
	1 Month	1.500 *	0.151	0.000	0.135 *	0.022	0.000
	6 Months	1.500 *	0.151	0.000	0.135 *	0.022	0.000

* The mean difference is significant at the 0.05 level.

**Table 5:** Bonferroni pairwise comparisons of the Upper Lip Length at Maximum Smile (millimeters) at different time intervals for the participating subjects (*n* = 100).

		Control Group			Test Group		
Time Intervals	Compared to;	Mean Difference	Std. Error	* Sig.	Mean Difference	Std. Error	* Sig.
Baseline	1 Month	−2.000	0.000		−2.000	0.000	
	6 Months	−2.000	0.000		−2.000	0.000	
	1 Year	−2.924 *	0.984	0.022	−1.840 *	0.037	0.000
1 Month	Baseline	2.000	0.000		2.000	0.000	
	6 Months	0.000	0.000		0.000	0.000	
	1 Year	−0.924	0.984	1.000	0.160 *	0.037	0.000
6 Months	Baseline	2.000	0.000		2.000	0.000	
	1 Month	0.000	0.000		0.000	0.000	
	1 Year	−0.924	0.984	1.000	0.160 *	0.037	0.000
1 Year	Baseline	2.924 *	0.984	0.022	1.840 *	0.037	0.000
	1 Month	0.924	0.984	1.000	−0.160 *	0.037	0.000
	6 Months	0.924	0.984	1.000	−0.160 *	0.037	0.000

* The mean difference is significant at the 0.05 level.

**Table 6:** Bonferroni pairwise comparisons of the Upper Lip Length at Rest (millimeters) at different time intervals for the participating subjects (*n* = 100).

		Control Group			Test Group		
Time Intervals	Compared to;	Mean Difference	Std. Error	* Sig.	Mean Difference	Std. Error	* Sig.
Baseline	1 Month	0.000	0.000		0.000	0.000	
	6 Months	0.000	0.000		0.000	0.000	
	1 Year	0.092	0.110	1.000	0.000	0.000	
1 Month	Baseline	0.000	0.000		0.000	0.000	
	6 Months	0.000	0.000		0.000	0.000	
	1 Year	0.092	0.110	1.000	0.000	0.000	
6 Months	Baseline	0.000	0.000		0.000	0.000	
	1 Month	0.000	0.000		0.000	0.000	
	1 Year	0.092	0.110	1.000	0.000	0.000	
1 Year	Baseline	−0.092	0.110	1.000	0.000	0.000	
	1 Month	−0.092	0.110	1.000	0.000	0.000	
	6 Months	−0.092	0.110	1.000	0.000	0.000	

* The mean difference is significant at the 0.05 level.

## Data Availability

Data is available on request from the corresponding author.
